# Advances in three-dimensional modeling of ischemic injury

**DOI:** 10.1177/0271678X251409022

**Published:** 2026-01-16

**Authors:** Aurora Halkoluoto, Hiramani Dhungana, Meike Hedwig Keuters

**Affiliations:** Neuroscience Center, HiLIFE, University of Helsinki, Helsinki, Finland

**Keywords:** Ischemia, ischemic events, stem cells, brain organoids, microfluidic devices, in vivo engraftment systems, 3D bioprinting

## Abstract

3D human stem cell-based brain models are capturing significant interest within the scientific community and general population. Up to date, a tremendous portion of study results cannot be translated to clinics due to poor study models, like over-simplified 2D cell-culture models or unfitted animal models. 3D brain models appear to bridge the gap between pre-clinical and clinical research improving drug discovery. This systematic review aims at investigating the rapid advancement and growing body of research involving 3D brain models, particularly organoids and assembloids. Our focus will be on the models’ application in the context of ischemic events. We aim at examining how these innovative models can enhance in vitro ischemia research, thus, increase the translational value of studies. We provide a brief overview of ischemic pathology and the limitations of current in vitro models. Next, we discuss corticogenesis, the fetal developmental process replicated by 3D brain models, and the progress made in generating physiologically relevant models. We further examine advanced 3D platforms, including *in vivo* engraftment systems, microfluidic devices, and 3D bioprinting, to develop vascularized organoid models for studying ischemic insults. Finally, we provide a critical, evidence-based analysis of the models’ potential and challenges, highlighting remaining hurdles that must be addressed.

## Ischemic stroke and in vitro study models

Disrupted blood flow to the brain, commonly referred to as ischemic stroke, can cause significant brain damage due to insufficient delivery of oxygen and nutrients and lead to disability and even death.^1^ Despite advancements in research, current treatment^2^ options remain limited and time sensitive. Most of the therapeutic compounds proven beneficial in in vitro or animal models of stroke have failed in clinical trials. Therefore, a model accurately recapitulating the pathophysiology of the human brain could significantly facilitate translational success. In this regard, human-derived 3D brain models present a promising platform for conducting mechanistic studies and for screening potential therapeutics for ischemic stroke.

Traditional cell culture models lack dimensionality, cellular diversity, and cell-cell and cell-matrix interaction. In contrast, 3D cell culture models allow cells to proliferate, differentiate, and maintain a microenvironment through cell-cell and cell-matrix interaction. Hence, they can be an invaluable tool for understanding both normal and pathophysiological processes in a physiologically relevant context. Numerous 3D cell culture models have been established utilizing anchorage-independent, hanging drop, magnetic levitation, and scaffold- or hydrogel-based technologies.^3^ Among the most promising 3D models are organoids, which are distinguished by their capacity for self-proliferation and self-organization. Brain organoids can be engineered from embryonic stem cells (ESCs) or induced pluripotent stem cells (iPSCs). They exhibit structural architecture and functionality comparable to the developing human brain. Thus, organoids offer a near-physiological model for studying mechanisms related to organ development or disease pathology.^4^ Furthermore, they serve as a promising tool for therapeutic drug screening for both traditional and personalized medicine.

This review will highlight distinct features of brain organoids and other 3D models, exploring their advantages as well as current limitations. Additionally, it will assess the models’ applicability for studying ischemic insults and their therapeutic capacity.

## Ischemia and ischemic stroke

**Figure 1.**
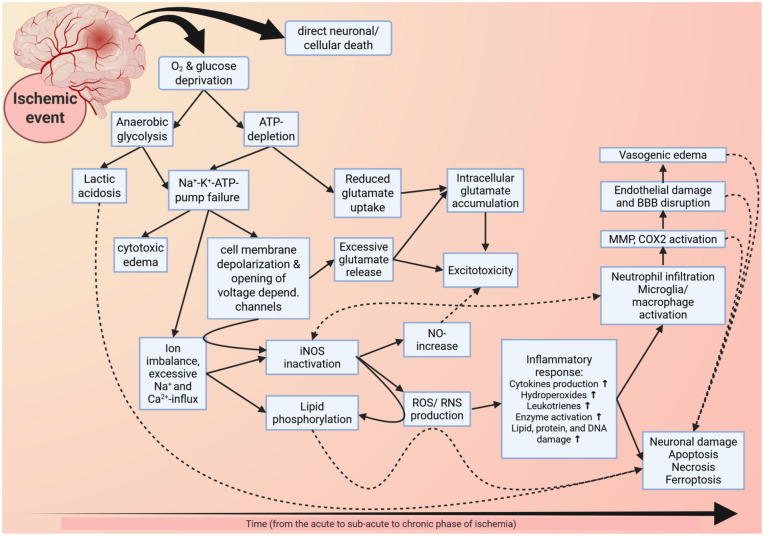
Figure 1 is a schematic representation of the cascades and processes initiated by ischemia (note that the image is not exhaustive). The displayed cascades and processes culminate in cell death, inflammation, and tissue damage, eventually causing neurological, somatosensory, and motor deficits. O_2_: oxygen; ATP: adenosintriphosphat; Na^+^: sodium; K^+^: potassium; Ca^2+^: calcium, iNOS: inducible nitric oxide synthase; NO: nitric oxide; ROS: reactive oxygen species, RNS: reactive nitrogen species; BBB: blood-brain barrier; MMP: matrix metalloproteinase, COX2: cytochrome c oxidase II; (adapted from Keuters, 2021).

Ischemic stroke results from blocked or severely reduced blood flow—caused by either a thrombus or embolus—and consequently impairs the supply of oxygen and nutrients to the affected area. Ischemic stroke may be transient, with partial or full reperfusion limiting tissue damage, or permanent causing extensive brain injury. However, even brief ischemic episodes can severely reduce blood flow triggering energy failure and a cascade of harmful processes. These include ionic imbalance, acidosis, excitotoxicity/ cytotoxicity, oxidative stress, blood-brain barrier (BBB) disruption, and immune cell activation and infiltration (Figure 1).^5^ In the early, acute post-ischemic phase, necrotic cell death predominates. In the secondary phase, inflammation and apoptosis contribute to extended cell death and tissue damage.^6^

Current acute treatment options include tissue plasminogen activator (tPA) and mechanical thrombectomy. However, both are limited by a narrow therapeutic window, with the latter inheriting the risks of mechanical intervention. As such, novel therapeutic strategies are needed to prevent continued ischemic brain damage and to promote long-term neurological and behavioral recovery in stroke patients.^2^ Despite promising preclinical data, many potential therapies have failed in clinical trials.^7^ These failures are largely due to several critical factors, including insufficient methodological rigor, limited sample size, and issues pertaining to external validity, like homogeneity and age of animal cohorts.^8^ Moreover, translation is further impeded by the absence of relevant comorbidities typically seen in patients, by genetic background variations among individuals, and by species-specific differences.^7,8^

## Two-dimensional in vitro stroke models

2D in vitro cell cultures have been used to model hypoxic-ischemic injuries. These 2D hypoxic ischemia models are crucial to understand specific pathophysiologic mechanisms underlying ischemic conditions like necrosis, apoptosis, and autophagy.^9^ Advantages are the models’ cost-efficiency, their suitability for high-throughput approaches, and the possibility to utilize various brain cell types, as well as the overall reduction of animal studies. However, 2D models cannot fully replicate the in vivo pathology of an ischemic insult because they lack the vasculature and the intricate interactions occurring within the CNS, and between the CNS and the periphery. For mimicking ischemic events in vitro, primary cell cultures, immortalized cell lines, and organotypic slice or brain slice cultures are commonly used. These cultures are deprived of oxygen and glucose (oxygen-glucose-deprivation model (OGD)), or cellular metabolic pathways are inhibited. A major disadvantage of brain slices is their short viability–typically around 12 h for rodent brain slices, and longer lifespan of acute organotypic slices (⩽24 h).^9^ Additionally, slice cultures show signs of damage from the process of slicing itself, and thus, immune cell activation.^10^ The limited availability and viability of primary cell lines or human tissue samples are another important limiting factor. Therefore, immortalized cell lines, such as glioblastoma-derived SH-SY5Y cells, have been widely used as an unlimited human cell source for stroke research.^11^

### The fetal stage of corticogenesis

The development of the fetal brain begins after the neural plate folds into the neural tube (_~_gestational week 4, GW 4). The dorsal telencephalon, containing a monolayered neuroepithelium composed of neuroepithelial cells with apical-basal polarity, eventually develops into the cerebral cortex.^12^ These neuroepithelial cells can divide in two distinct ways: symmetrically to self-renew, or asymmetrically to produce (i) radial glia cells (RG) in the ventricular zone (VZ) and (ii) neurons in the outer layer.^12,13^ RG are bipolar and can form both apical and basal processes. In addition to direct division toward neurons, RG can produce neurons indirectly via a neural progenitor cell (NPC) pool called intermediate progenitor cells.^14,15^ Intermediate progenitors delaminate in the subventricular zone (SVZ) basally from the VZ (GW 7-8).^16,17^ The fate of these cells can lead either to proliferation, generating additional intermediate progenitors for cortex expansion, or to neurogenesis, producing more differentiated neurons.^14,15^ In humans, the SVZ is subdivided into outer and inner parts. The inner SVZ primarily contains intermediate progenitor cells. The outer SVZ is characterized by the presence of specific outer RG (oRG), which share many properties with traditional RG. Both cell types are connected to the basal lamina, have basal processes, and express the PAX6 transcription factor.^16,17^ They can also divide asymmetrically, generating one oRG-daughter cell and another intermediate progenitor cell. During this developmental process, two distinct zones, the cortical plate and the intermediate zone, are evident.^13^ The cortical plate consists of young neurons divided from RG, oRG, or intermediate progenitors.^14–18^ The intermediate plate starts to develop between the cortical plate and SVZ, consisting of both multi- and unipolar neurons.^13^ During corticogenesis, distinct NPC populations—such as RG, oRG, and intermediate progenitor cells—generate neurons that migrate to the cortical plate forming the basis of the multilayered structure of the cerebral cortex.

### Development and progress in brain organoid research

In 2006, Yamanaka’s laboratory made a groundbreaking discovery by reprogramming mouse embryonic and adult fibroblasts to iPSCs using four key transcription factors (Oct3/4, Sox2, c-MYC, and Klf4).^19^ Shortly after, a similar protocol to reprogram human skin fibroblasts to iPSCs was published.^20,21^ iPSCs are versatile as they can differentiate into cells from all three germ layers, including neurons, endothelial cells (ECs), and microglia. Since early on, neurospheres and neural aggregates, entirely made of neural stem and progenitor cells, have been used for simple 2D and 3D culture systems. While these models lack the complexity of the human brain (cellular composition and organization), they paved the way for developing more sophisticated cerebral organoid models. The initial breakthrough in developing neuronal organoids was reported by Eiraku et al. in 2011. They utilized a serum-free floating culture of embryoid body-like aggregates, known as the quick aggregation (SFEBq) method. This approach allowed ESCs to differentiate into neuroectoderm-like epithelium and to form cortical neurons in a self-organizing manner, mimicking processes observed during early corticogenesis.^22^

Lancaster et al. developed the first precise method for creating brain organoids by leveraging the self-organizational properties of pluripotent stem cells and embedding them in an artificial matrix, so-called Matrigel.^4,23^ The resulting cerebral organoids exhibited distinct brain regions, including the cerebral and prefrontal cortex, ventral forebrain, retina, choroid plexus, and hippocampus. Importantly, cerebral cortex-like structures in the organoids resemble the spatial organization of the developing human cortex. As such, self-organizing brain organoids represent a fetal stage of corticogenesis (as described previously). Long-term maturation of these organoids (⩾10 months) revealed functional neuronal calcium oscillation.^4^ While the minimal use of growth factors and small molecules in this unguided protocol indicates a high level of self-organization, it also causes heterogeneity between cerebral organoids.^24^ The observed heterogeneity led to the development of guided protocols using specific growth factors to direct organoid differentiation to a specific brain region, such as the cerebellum, midbrain, striatum, or cortex.^25^

The common features of various cortical organoid protocols are the dual SMAD inhibition for neuronal induction and the variation of growth factors, for example, BDNF, GDNF, or NT3, supporting neuronal maturation.^26–28^ This results in organoids with cortical layering corresponding to the third trimester in human brain development.

**Figure 2.**
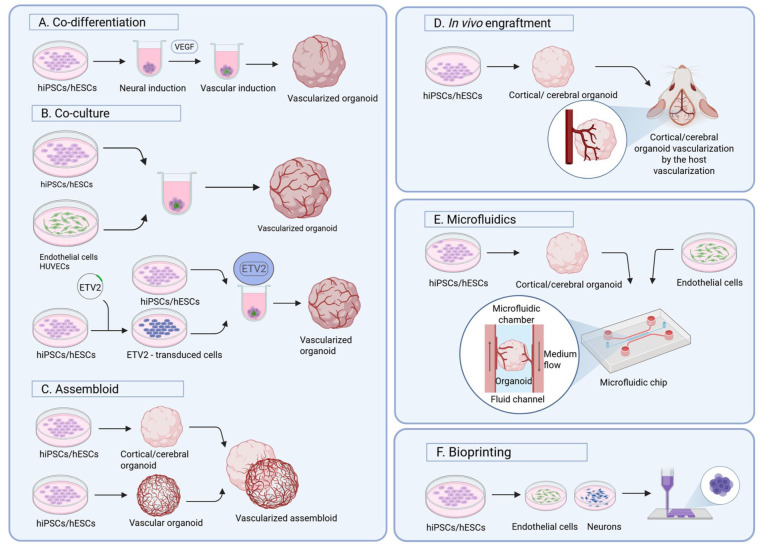
This schematic image shows currently prominent strategies for generating vascularized 3D brain cell cultures. (a) In the co-differentiation or multi-lineage approach human iPSCs or ESCs are differentiated toward neuronal or vascular cell fates by concurrent addition of small molecules. (b, part I) In co-culture protocols, human iPSCs/ ESCs that have been differentiated in parallel—but in separate cultures—toward neurons and endothelial cells, are combined to form organoids. HUVECs may be used as an alternative source to endothelial cells. (b, part II) Human iPSCs/ESCs, differentiated toward neurons, are co-cultured with ETV2-overexpressing cells to form vascularized organoids. (c) In the assembloid approach, separately generated brain and vascular organoids are fused to form the assembloid. (d) For the in vivo engraftment, brain organoids are matured before engrafted to the cerebrum of immunodeficient mice. (e) Microfluidic devices typically consist of two or more chambers, mostly separated by porous membranes, enabling fluid flow and potentially cell migration. Neuronal cells (or even organoids) can be seeded into one chamber and endothelial cells (or vascular progenitor cells) into connecting or adjacent chambers. (f) For 3D bioprinting approaches, stem cell-derived neuronal, endothelial, and glial cells are printed separately in predefined spatial patterns to construct vascularized cerebral tissues. (Created with BioRender.com.) hiPSC: human induced pluripotent stem cell; hESC: human embryonic stem cell; VEGF: vascular endothelial growth factor; ETV: ETS variant transcription factor 2.

Assembloids are created by fusing two or more distinct organoids, each modeling a single specific brain region, enhancing both structural and functional complexity (Figure 2(c)). For instance, combining dorsal and ventral forebrain spheroids into assembloids facilitates the migration of ventral cells into dorsal domains.^29^ Similarly, three-part cortico-motor assembloids were merged from cortical, motor, and spinal cord spheroids. Stimulation of the cortical spheroid caused muscle contractions within the motor spheroid, mediated by the spinal cord spheroid.^30^

A major limiting factor in brain organoid research has been the insufficient delivery of oxygen and glucose to deeper organoid layers, hindering dimensional growth and maturation. This was partially circumvented either by slicing organoids—reducing cell death and increasing maturation^31^—or by engrafting them into the rodent brain—promoting neuronal circuit formation.^32^ The latter method is also relevant for the vascularization of organoids.

## Vascularization of brain organoids

Functional vasculature is critical for delivering oxygen and nutrients to maintain cellular and metabolic homeostasis. Many attempts to create a functional vasculature within brain organoids have been reported. Fundamental strategies include co-culturing iPSCs or neural organoids with ECs or aggregates (Figure 2(a)–(c)),^33–36^ engrafting brain organoids into host animals (Figure 2(d)),^34,35,37^ or using microfluidic devices (Figure 2(e)).^38^

In vitro generation of vascularized organoids with functional vasculature has employed multiple strategies, including multidirectional differentiation, genetic engineering, and co-culture systems. One prominent approach is multi-lineage differentiation. Ham et al. demonstrated the formation of blood vessel-like structures in organoids using vascular endothelial growth factor (VEGF; Figure 2(a)), albeit with a partial compromise in neuronal identity.^39^ Most commonly, vascular brain organoids are generated by co-culturing vascular cells or vascular organoids and neuronal progenitors or neuronal organoids. As such, Shi and colleagues created vascularized cortical organoids by co-culturing human umbilical vein endothelial cells (HUVECs) and human iPSCs. These organoids displayed cytoarchitecture and gene expression patterns similar to the human fetal telencephalon.^35^

Pham et al. reported vascularization of cerebral organoids when introducing iPSC-derived ECs into organoids after 34 days of development by re-embedding them in Matrigel (Figure 2(b), co-culture part I).^34^ Similarly, Wörsdörfer and colleagues created vascularized neural organoids by merging neural and mesodermal aggregates.^40^ Cakir et al. co-cultured genetically modified hESCs, which ectopically express the ETV2 gene (ETS Variant Transcription Factor 2), with iPSCs (Figure 2(b), part II).^33^ ETV2-overexpressing cells form vessel-like structures with BBB characteristics. These organoids also show typical cortical architecture, reduced apoptosis, and FITC-Dextran uptake, indicating functionality of the vessel-like structures.^33^ Additionally, overexpression of NEUROD1 has been shown to enable the formation of neurovascular organoids retaining both neural and vascular function for up to 45 days.^41^ Taking cues from research, including fusion of assembloids and the vascular organoid development protocol,^42^ Kook et al. assembled cerebral brain organoids from vascular spheroids containing HUVECs, human dermal fibroblasts, and human umbilical cord blood-derived mesenchymal stem cells. These assembloids developed a vascular core with sprouting vessel-like structures and more mature neuronal structures including astrocytes.^43^ In a similar fashion have assembloids been generated by fusing cerebral organoids with brain-specific vascular organoids exhibiting key BBB characteristics.^36,44^ Notably, the addition of microglia to these assembloids enabled immune responsiveness, leading to synaptic pruning, and an increase in neural progenitor cells.^45^

## Vascularization through in vivo engraftment

In vitro modeling of vascular organoids can produce capillary-like structures. However, to achieve full functionality and to mimic in vivo/ physiological conditions, liquid flow for nutrient and oxygen transport into the organoids is needed. Currently, this has only been achieved by engrafting organoids into host tissues (Figure 2(d)). Indeed, several studies demonstrated in vivo organoid-vascularization. For the first attempt to vascularize human cerebral organoids, immunodeficient NOD/SCID mice were used.^37^ Functional circulation of engrafted organoids was confirmed by ex vivo imaging after dextran perfusion.^33^ Further studies have shown vascularization, better survival, and improved maturation of engrafted organoids into the mouse model.^46,47^ The drawback of these studies is the presence of murine capillaries vascularizing the organoids instead of human endothelial vasculature, as mouse-derived vasculature was shown to integrate with cerebral organoids. For example, cortical organoids containing ETV-2 induced capillary structures were implanted into the hind limb of Rag2 and GammaC mice to promote functional vascularization of brain organoids.^33^ To address this, researchers have attempted to develop human-specific vasculature capable of integrating with murine vessels. Pham et al. engrafted organoids with iPSC-derived ECs into NGS mice (NOD scid γ), indicating increased organoid survival.^34^ Similarly, Shi et al. engrafted organoids with HUVEC-derived ECs into the S1 cortex of NOD/SCID mice and observed the formation of functional, perfusable vascular networks. The vasculature supported enhanced survival, gliogenesis, and synaptic connectivity in 40–50-day-old organoids.^35^

Taken together, engrafted brain organoids can achieve functionality. However, the presence and infiltration of host vascular, immune, and other non-resident cells into organoids can create a complex environment, making it hard to deduce sufficient conclusion for the experiment.

## Microfluidic devices

Microfluidic devices or organ-on-chip devices allow for precise microscale-perfusion of liquids into cells, tissues, or organoids (Figure 2(e)) mimicking physiological blood flow. As they present a suitable microenvironment with controllable growth conditions, they enable vascular network development with BBB characteristics.^48^

The Transwell system, a simple microfluidic device, consists of one chamber for brain cells and one for ECs separated by a porous membrane. While useful for studying BBB integrity,^49^ the system lacks dynamic flow and shear stress. A more advanced microfluidic brain-on-chip model enables fluid flow and supports the formation of micro-vessels in the presence of ECs and pericytes.^50^ The advantage of these chips is the possibility to model physiologically relevant BBB characteristics mediated by different cell types, including neurons, astrocytes, ECs, and pericytes.^51,52^ Despite advancements, these chips fall short in accurately mimicking the 3D architecture of brain tissue.^53^

Stanton and colleagues translated their integral co-culture model, miBrain, which mimics key features of the human brain, including structural organization, functional networks, and the BBB,^54^ into a brain-on-chip model. As before, six major brain cell types, namely iPSC-derived neurons, microglia, astrocytes, oligodendroglia, pericytes, and brain microvascular endothelial cells were seeded along with human fibroblasts in neuromatrix hydrogel.^54,55^ Importantly, a special technique of micro-dissecting the outer edge of 3D hydrogel-cultures enabled diffusion to the chip’s functional vessel system. The resulting vessels reach an average diameter of 13.6 µm, with branching characteristics similar to those in vivo.^55^

Salmon and colleagues used microfluidic devices and 3D brain organoids to mimic the architecture of the fetal brain. They designed a 3D-printed chip featuring a large central chamber holding brain organoids, flanked by channels lined with pericytes and ECs. This setup enables the formation of capillary-like vessels sprouting toward a 6-day-old cerebral organoid to form perfusable vasculature on the organoid’s surface.^38^ So far, 3D microfluidic chips have been used to, for example, model prenatal cannabis exposure,^56^ or neonatal wrinkling of the brain.^57^ Microfluidic systems appear to enhance organoid maturity and reduce necrotic cores.^48^ As microfluidic technologies become more accessible, further advancement in brain organoid vascularization is expected.

Is there a lesson to learn from non-cerebral organoid models?

Using microfluidic systems to vascularize non-cerebral organoids was highly successful.^58,59^ Homan and colleagues promoted vascularization of kidney organoids by developing a microfluidic system supported by extracellular matrix (ECM).^58^ Similarly, vascularization was seen in liver organoids after co-culturing adult stem cell-derived liver organoids with ECs and decellularized liver ECM under microfluidic flow.^59^ However, it remains unclear how to translate these approaches to brain organoids.

## 3D organoids and their relevance in ischemic stroke research

Cerebral and cortical organoids and assembloids have been used to study ischemic stroke in vitro using, for example, the OGD model.^2,11^ For in vitro OGD models, hypoxic conditions (1%–3% O₂) are typically maintained for hours to days, depending on the experimental aim, as extended hypoxia induces substantial cell death.^60^ Reoxygenation of oxygen-glucose-deprived cultures acts as a relevant model for ischemia-reperfusion injury (IRI), and has been reported to cause neuronal cell death, impaired neuronal complexity, and loss of cortical stratification in cerebral and cortical organoids.^60–62^ Especially, intermediate and neuronal progenitor cells seem to be affected by the OGD/reoxygenation (OGD/R) model.^60^ While OGD (OGD/R) is the most common model, hypoxia or hypoxia/reoxygenation, is also a commonly used model for ischemic insults (compare Table 1). Paşca et al. showed that the death of progenitor cell populations might result from an activation of the endoplasmic reticulum stress-response pathway, a pathway involved in protein homeostasis and apparently in cortical development.^63^ De Paola and colleagues demonstrated hypoxia-induced cytotoxicity in cerebral organoids.^61^ And Iwasa et al. have linked the fatty-acid-related peroxisome proliferator-activated receptor (PPAR) signaling pathway and the pyruvate kinase isoform M2 (*PKM2*) to OGD/R.^64^

**Table 1.** Comparative table of published human-derived 3D brain cell models of ischemic events.

3D cell culture model	Study model	Tested compound(s)	Findings	Advantages	Disadvantages	Author(s)
Brain organoids, up to 6 months of age, H9 hESCs	Neonatal hypoxic injury (NHI):Hypoxia at day 10: 1% or 8% O2 for 72 h to 35 days, ±minocycline; post-hypoxia: organoids were kept for up to 6 months	Minocycline	Hypoxia repressed gene markers for forebrain, oligodendrocytes, glial cells, cortical layers, genes important for migration of cortical neurons; ventral markers were unaffected or increased; minocycline attenuated the negative hypoxic effect on dorsal brain, oligodendrocytes, and neuronal progenitor genes	Avoiding matrigel decreased risk of contamination, lower cost, simplified imaging, and no unknown quantities of growth factors that copurify with the product, during production, exogenous growth factors weren’t needed, no necrosis issues during the first 5–6 months observed	Neonatal hypoxic model, not a direct ischemic stroke model; only hypoxia, no nutrient restriction; devoid of vasculature, use of minocycline ► can decrease microglia-activation (MG might be actual target);	Boisvert et al., 2019^65^
Cortical organoids, hiPSC lines	Hypoxia/R: <1% O2 for 48 h ► 21% O2 for 24, 48, or 72 h	ISRIB	Reduction of specific cortical progenitors (TBR2-pos. cells, contributing to cortex expansion), preventable by pharmacological modulation of the unfolded protein response (UPR); the UPR modulator ISRIB prevents hypoxia-induced TBR2 defects	Measurements of *P*O2 were done at organoid-surface and -center; tested differentially expressed genes (focus on hypoxic response); more mature organoids then average (75 days of age); result validation in primary human cortical tissue (in vitro); model of either hypoxic encephalopathy of prematurity, or second-trimester placental insufficiency;	Not intended as a model of ischemia; no glucose-deprivation; devoid of immune and vascular cells;	Pașca et al., 2019^63^
Cerebral forebrain organoids, hESCs	Prenatal hypoxic injury (HI)- transient hypoxia: 3% O2 for 24 h ► reoxygenation (21% O2) until analyses	None	Immediate and prolonged apoptosis in cerebral organoids ► especially in ORG, differentiating neuroblasts ►definition of vulnerable window of neural differentiation;	Confirmed division mode changes in NPCs; immediate and long-term effects of hypoxia were studied; included isochronic cohort studies of NPCs;	Not intended as an ischemic stroke but prenatal hypoxic injury-model; immature organoids (28–42 days of age); focus on differentiating cells;	Daviaud et al., 2019^60^
Multicellular 3D neurovascular unit organoid,	Modeling effects of hypoxia and neuroinflammation on BBB function: 0.1% O2 for 24 h	Ecoisolariciresinol diglucoside and 2-arachidonoyl glycerol	Hypoxic conditions triggered: increased permeability, pro-inflammatory cytokine production, and increased oxidative stress; anti-inflammatory compounds reversed neuroinflammation;	Big variety of cell types: brain microvascular endothelial cells, pericytes, astrocytes, microglia, oligodendrocytes and neurons ► BBB characteristics; investigation of ROS and neuroinflammation and BBB function; disrupted tight junctions under hypoxia;	Focus was on exploring mechanisms that lead to the disruption of the BBB;	Nzou et al., 2020^66^
Cerebral Organoids, hiPSCs	OGD/R: 1 h at 1% O2 in glucose-free medium ► 1 h reoxygenation in growth medium	None	After OGD/R: downregulation of AHSG, TTR, FGG; no significant change of FLT; upregulation of PKM2	Study results on organoids were compared with 2D human and murine cell cultures; ensured the insult was ischemic and not a postnatal metabolic shift;	Immature organoids (42 days of age); focus was only on neural cells; organoids were devoid of other cell types;	Iwasa et al., 2021^64^
Neural organoids, hiNSC lines	hypoxia-reoxygenation model: 1% O2 for 48 h; reoxygenation: 21% O2 for 24 h	None	Hypoxia caused neuronal damage; reoxygenation restored neural cell proliferation; neuronal maturation remained impaired	Older/mature organoids , 85 days of age; hypoxia, glucose-deprivation, and ODG were tested/ compared; suitable to study development post-hypoxia	Devoid of vasculature; devoid of immune cells; no cortical circuits;	Kim et al., 2021^62^
Neurospheroids, hiPSCs or hESCs	OGD: 6 hours on 1- or 4-week-old organoids (0.0%–0.5% O2 & glucose-free cNEMedia –> luminescent measurement as a high-throughput read-out (24 h OGD was tested too)	Z-VAD-FMK	Established the method of detecting luminescent signals as an indicator of suffering from OGD and proved it was due to hypoxia-induced cytotoxicity; apparently, 4-week-old spheroids respond better then 1-week old; treating spheroids with Z-VAD-FMK was not	Comparison of develop. early organoids; stable introduction of firefly luciferase reporter via lentiviral vector transduction; luminescent is simple for longitudinally follow-up of growth and viability; size and differentiation were highly reproducible;	1- or 4-week-old organoids ► very young; not organoids, NPC-only spheroids, devoid of astrocytic, microglial, and vascular cells; diameter >400 µm, high probability of necrotic core; 1-week old spheroids recovered almost completely from OGD; the neuroprotective effect of Z-VAD-FMK was not reflected in the spheroid-OGD model;	Van Breedam et al., 2022^67^
Cerebral organoids, hESCs	OGD: <1% O2, glucose-free medium	Z-VAD-FMK, navitoclax, edaravone, butylphthalide, ZL006, P7C3-A20	Aim was to establish an in-vitro humanized ischemic stroke model of OGD and to explore its application in testing anti-stroke drugs; models’ sensitivity to OGD was proven, low oxygen induced hypoxic injury in a OGD exposure time-dependent manner	Comparison of organoids to achieve consistency on cell-compositions and diameters ► decreased variability; time-effect relationship of OGD was explored ► experimental reference for selecting the proper OGD time and ischemic injury degree; efficacy tested using pro- and anti-apoptotic drugs;	Chosen organoid model and/or size show spontaneous signs of necrosis/necrotic cores; devoid of vasculature; devoid of microglia, only few astrocytes (spontaneous);	Wang et al., 2023^68^
Cortical organoids; hiPSCs	OGD: 2 h or 8 h at 0.1% O2, deoxygenated glucose-free medium; collection: 0, 24, 48 h	None	OGD-exposure time-dependent changes of LDH release, impaired neuronal network complexity, neuronal death, increased GFAP reactivity,	Showed neuronal-glial interactions and connectivity; determined astrocytic response to OGD;	Immature organoids (60 d of age); devoid of microglia; devoid of vasculature; apparently a necrotic core; specific OGD-mediated toxicity in the outer layers, but not in the core;	De Paola et al., 2023^61^
Cerebral organoids, hiPSCs or hESCs	Photothrombotic stroke model; 1-week post-ischemia: 1, 3 organoids, or dissociated cells from 3 organoids were transplanted to the junction of infarct & peri-infarct zone	None	Cells of engrafted organoids differentiated into target neurons which sent axons into distant brain areas, integrated to host neural circuit ► possibly improving sensorimotor deficits and repairing tissue; injected single cells failed to repair infarcted tissue	Mixture of in vitro and in vivo might hold translational advantages; recovery from stroke can be studied/boosted	No in vitro hypoxia or OGD experiments; focused on recovery not on effects of OGD/ hypoxia	Cao et al., 2023^69^
Brain organoids, hiPSCs or hESCs (and MCAO mouse model)	OGD on human brain organoids: 37 °C, less than 1% O2, 5% CO2, 70% humidity for 0–24 h, in glucose-free medium; middle cerebral artery occlusion (MCAO) in mice	Traditional Chinese Medicine (TCM): DengZhan-ShengMai capsule	Identification of astrocyte-type leading to hyperplasia; increased numbers of astrocytes type 1, 2, and 3; decreased numbers of cortical excitatory neurons and neural stem cells in vitro; MCAO mouse model: dysfunction of neural cells, downregulation of several neural genes; in vitro and in vivo: cortical excitatory neurons: apoptosis and aging.	Back-to-back investigation of mouse stroke and organoid OGD ► direct comparison of in vitro and in vivo results; in vitro organoid models allow deeper study of the molecular characteristics and pathological processes; combination of organoid model with multi-omics strategy; TCM was proven to be protective, both in vitro and in vivo;	Immature organoids (40 days of age); apparently devoid of glial cells; devoid of vasculature;	Zhu et al., 2025^70^

hESC: human embryonic stem cell; NHI: neonatal hypoxic injury; hiPSC: human induced pluripotent stem cell; Hypoxia/R: hypoxia/ reperfusion; UPR: unfolded protein response; HI: hypoxic injury; NPC: neural precursor cell; OGD/R: oxygen – glucose deprivation/reoxygenation; MCAO: middle cerebral artery occlusion; TCM: Traditional Chinese Medicine.

Mechanistic studies aside, Wang et al. verified the sensitivity of their cerebral organoids to ischemic injury using pan-Caspase inhibitor Z-VAD-FMK and Bcl-2 inhibitor navitoclax. They further confirmed the efficacy of different neuroprotective compounds, namely edaravone, butylphthalide, P7C3-A20, and ZL006.^68^

Brain organoids are also being explored as a source for neuronal replacement and tissue regeneration after injury. Early studies in mice demonstrated that engrafting brain organoids improves overall survival compared to transplanting dissociated neurons as a single-cell suspension.^46^ Likewise, Wang et al. have shown that engrafting cerebral organoids after middle cerebral artery occlusion in rats promotes intrinsic neurogenesis and angiogenesis, synaptic construction, axonal regeneration, and migration of neurons to other brain areas (Table 1).^71^

Despite these advances, the absence of (human) vasculature and resident immune cells in current organoid models remains a major limitation in modeling ischemic stroke.

## Comparative models in ischemic stroke research

3D brain organoid models with functional vasculature (at best including neurovascular units, NVU) would be ideal for modeling ischemic stroke pathology and screening potential therapeutics. Nzou and colleagues published a protocol for NVU-organoids containing neurons, ECs, pericytes, microglia, and oligodendrocytes (Table 1).^72^ When applying the OGD model to their organoids, they detected neurotoxicity, neuroinflammation, oxidative stress, and signs of OGD-mediated BBB disturbance.^66^ However, the interpretation of the study results requires great caution as the model’s capillaries were non-functional.^66,72^

## 3D bioprinted models and their application in ischemic stroke

Lately, 3D bioprinting of human brain tissue (Figure 2(f)) has undergone great progress. Yan and colleagues successfully 3D bioprinted functional neural networks. They further succeeded in modeling Alexander’s Disease in 3D bioprinted brain tissue.^73^

While the focus is currently on genetically determined or mutation-based diseases, like Alexander’s, Alzheimer’s Disease, or Parkinson’s Disease, 3D bioprinted models to study ischemic stroke are under development. Examples include NVUs, bioprinted using a computer-based template,^74^ or models bioprinted from primary rat brain cells using Matrigel and alginate/collagen gels to replicate human ECM.^75^ The latter demonstrated a printing resolution of ~10 µm and tight junctions, formed by mouse endothelial and astroglial cells. Exposing the NVU-model to OGD induced ischemic stroke-related pathology, including decreased neuronal axon length and cell proliferation rate, fragmented vascular structures, disrupted tight junctions, increased LDH, and apoptosis markers.^76^ Yet, 3D bioprinting of brain tissue needs further optimization, for example, in printing resolution, bio-ink composition, and functionality. Another pitfall—the reliance on animal-based ink components to mimic ECM—needs to be addressed by improving synthetic polymers and/or scaffolds to support, for example, neural tube morphogenesis, cell survival, and differentiation.

## Discussion and conclusion

While 3D brain organoid models have improved immensely over the last few years, their application for studying ischemic events remains challenging.

Patient-derived iPSCs can be efficiently engineered into functional brain organoids or assembloids, enabling the modeling of genetically driven neurological or neurodevelopmental diseases. While risk factors for ischemic stroke may have genetic underpinnings, the condition itself is rarely just genetical. At best, it is understood as an acute cerebrovascular event rather than a progressive disease. When modeling cerebrovascular events driven by blood flow obstruction, the absence of functional vasculature in organoids and assembloids hinders accurate replication of ischemic events and thus, interpretation and reliable translation of test results. Yet, models mimicking oxygen and glucose deprivation, or hypoxic injury—key factors in stroke pathology—can give valuable insights into basic processes, for example, on the level of cells, cellular networks, genes, cytokines, or transcription factors (Table 1).^61–64, 67,70^ Many of these studies included drug testing of known compounds as positive controls. Indeed, the majority proved to have an effective 3D cell culture model for hypoxia, hypoxia-R, or OGD.^63,66,67,68^ However, most of the models develop necrotic cores, which need to be carefully considered when interpreting test results.

Besides the lack of functional vasculature in current models, most of them are additionally devoid of immune cells, such as microglial cells. However, this appears problematic when modeling a condition like ischemic stroke, which is characterized by a strong immune cell response, contributing to tissue damage, especially during the secondary phase of IRI. Thus, modeling ischemia in current organoid models, largely devoid of immune cells, remains superficial, with limited translational relevance. While incorporating glial cells into organoids allows partial mimicry of post-ischemic immune responses, forming functional capillaries—let alone a complex, fully integrated vascular system—remains unresolved.

While the first miBrain model presented by Stanton et al. neither fell into the category of organoids, nor the category of microfluidic devices, the approach was yet promising in the context of advancing immunocompetence in culture models.^54^ More importantly, however, their brain-on-chip^55^ presents a highly suitable model to studying ischemic insults.

It may therefore be anticipated that using microfluidic devices or 3D bioprinting will ultimately outperform organoid-based models for studying ischemic events. Both microfluidic devices and 3D bioprinted brain structures hold the potential to be readily seeded, bioprinted respectively, from neuronal, glial, and vascular cells. The arrangements of the chambers of microfluidic devices enable instant nutrient and oxygen supply, sheer stress, etc. Vascular cells have even been suggested to be bioprinted using a rigid bioink-scaffold into tube-like structures.

Furthermore, most ischemic events are accompanied or even caused by one or more pre-stroke incidences or comorbidities, such as atherosclerosis, diabetes, high blood pressure, amyloid-beta plaques. Also, transient ischemic attacks (TIA) are often harbingers of a stroke. Certain neurodegenerative diseases or age-related comorbidities could be modeled in vitro, for example, by using AD patient-derived iPSC lines, or by aging the organoids. Other comorbidities, such as high blood pressure, appear to be more complicated. However, comparable and potentially transferable 3D in vitro cultures, which have a slightly different scope, have been established. As such, Liang et al., were able to reproduce blood pressure, shear stress, and circumferential strain in an endothelial cell culture system (ECCS) containing a microfluidic chip.^77^ Further existing models, like the model for atherosclerosis in endothelialized tissue-engineered blood vessels (TEBVs)^78^ or the lipopolysaccharide-mediated vascular inflammation model^79^ might be introduced to or combined with brain organoids to implement comorbidities to ischemic injury-organoid models.

Organoid development has always been challenged by a seemingly ever-present and unwanted organoid-to-organoid variability. This variability appears to emerge from various factors, such as self-directed organoid protocols, cell line variations, different cell passages used for seeding, or even matrigel batch-variations.^80–82^ While big variations might decrease the model’s applicability and interpretational value, a certain heterogeneity might have its value when thinking of, for example, recapitulating patient-to-patient and stroke-to-stroke differences.^80^ Nevertheless, huge effort has been put into reducing organoid culture variability. Among those are controlling embryoid body (EB) formation and organoid size, restricting the use of ROCKi,^82^ or characterizing the cell lines used.^63^

Important when planning 3D cell culture experiments using OGD/ OGD-R, or hypoxia/hypoxia-R, is choosing the best endpoint. Therefore, it is of utmost importance to consider the intended outcome and the chosen read-outs, for example, cell viability, metabolism, or pathway activation, as well as the choice of the 3D cell culture model itself. The age, or maturation of the organoid is a crucial variable as, for example, the state of differentiation of neural cells influences their resilience and response to hypoxic stimuli.^60^ Another important aspect is the size of the organoid/assembloid. The larger the model, the more extended the necrotic core might be. This also leads to higher inner-organoid ρO_2_-gradients, which possibly de- or accelerate any applied hypoxic condition. Thus, measuring the pO_2_ (for setting up the model as well as for data interpretation) would add value to the interpretation of the test results and potentially help with establishing the best experimental parameters. Overall, it appears wise to not only chose one timepoint, but rather an early (minutes to ~6 h), an intermediate (~6–24 h), and a late (~24–72 h) timepoint, to asses metabolic failure as well as cell death mechanisms.^61,63^

As a final remark, we want to point out that cerebral organoids and assembloids are a great study-model for various diseases. They are efficient, improve translatability, and present a major step toward a reduced use of animals in research. However, these models face challenges that must be addressed to better replicate human brain regions and maturation. Incorporating a broader variety of cell types beyond neurons, along with ECM components, would further enhance model fidelity. We conclude, microfluidic devices and 3D bioprinted brain tissues may currently offer a more suitable platform for ischemic stroke research.
